# From Marrow to Bone and Fat: Exploring the Multifaceted Roles of Leptin Receptor Positive Bone Marrow Mesenchymal Stromal Cells

**DOI:** 10.3390/cells13110910

**Published:** 2024-05-24

**Authors:** Parash Prasad, Jose A. Cancelas

**Affiliations:** Dana-Farber Cancer Institute, Harvard Medical School, Boston, MA 02215, USA; parash_prasad@dfci.harvard.edu

**Keywords:** bone marrow, mesenchymal stromal cells, leptin receptor, hematopoiesis, osteogenesis, adipogenesis

## Abstract

The bone marrow (BM) stromal cell microenvironment contains non-hematopoietic stromal cells called mesenchymal stromal cells (MSCs). MSCs are plastic adherent, form CFU-Fs, and give rise to osteogenic, adipogenic, chondrogenic progenitors, and most importantly provide HSC niche factor chemokine C-X-C motif ligand 12 (CXCL12) and stem cell factor (SCF). Different authors have defined different markers for mouse MSC identification like PDGFR^+^Sca-1^+^ subsets, Nestin^+^, or LepR^+^ cells. Of these, the LepR^+^ cells are the major source of SCF and CXCL12 in the BM microenvironment and play a major role in HSC maintenance and hematopoiesis. LepR^+^ cells give rise to most of the bones and BM adipocytes, further regulating the microenvironment. In adult BM, LepR^+^ cells are quiescent but after fracture or irradiation, they proliferate and differentiate into mesenchymal lineage osteogenic, adipogenic and/or chondrogenic cells. They also play a crucial role in the steady-state hematopoiesis process, as well as hematopoietic regeneration and the homing of hematopoietic stem cells (HSCs) after myeloablative injury and/or HSC transplantation. They line the sinusoidal cavities, maintain the trabeculae formation, and provide the space for HSC homing and retention. However, the LepR^+^ cell subset is heterogeneous; some subsets have higher adipogenic potential, while others express osteollineage-biased genes. Different transcription factors like Early B cell factor 3 (EBF3) or RunX2 help maintain this balance between the self-renewing and committed states, whether osteogenic or adipogenic. The study of LepR^+^ MSCs holds immense promise for advancing our understanding of HSC biology, tissue regeneration, metabolic disorders, and immune responses. In this review, we will discuss the origin of the BM resident LepR^+^ cells, different subtypes, and the role of LepR^+^ cells in maintaining hematopoiesis, osteogenesis, and BM adipogenesis following their multifaceted impact.

## 1. Introduction 

Hematopoiesis is a lifelong process where hematopoietic stem cells (HSCs) and progenitors (HSPCs) produce a continuous output of billions of blood cells per hour. After birth, this process mainly occurs within the bone marrow (BM) [1]. The process of hematopoiesis is regulated by cell intrinsic factors and non-cell autonomous microenvironmental factors. The microenvironment contains different cell types such as fibroblast-like stromal cells, osteoblasts, adipocytes, pericytes and nerve cells [2]. The BM microenvironment also contains some special types of fibroblast-like cells, known as mesenchymal stromal cells (MSCs) that are capable of producing all tri-lineage cells (adipocytes, chondrocytes and osteoblasts) after intra-femoral transplant through injection, or in proper in vitro conditions [3]. Further, they secrete different niche factors like the chemokines stem cell factor (SCF) and C-X-C motif ligand 12 (CXCL12), angiopoietin, and Interleukin-7 (IL-7), which are essential for the regulation of hematopoiesis. 

The first description of BM MSC came from the German pathologist Cohnheim in 1868 [4,5], who explained that BM contains fibroblast-like cells that helps in wound healing. The work of Russian scientists Maximov and Friedenstein showed that BM stromal cells can help in hematopoiesis [6,7], and Caplan found that these cells can differentiate into osteoblasts, chondrocytes, myoblasts, and adipocytes in vitro, coining the name “mesenchymal stem cells” [8]. In 2005, the International Society for Cellular Therapy recommended the name “Multipotent Mesenchymal Stromal Cells” for these fibroblast-like plastic adherent cells with multi-lineage differentiation capacity [6]. In the last few years, several reports have characterized BM MSC phenotypically and functionally [9]. Initially, they were identified based on the fibroblast-colony-forming unit (CFU-F) forming ability and the expression of platelet-derived growth factor receptor-α (PDGFRα) [10]. However, we now know that BM MSCs represent a highly heterogenous population with the ability to express different levels of paracrine-acting cytokines and chemokines. The first evidence came from the analysis of CXCL12 locus knock-in reporter mice, which revealed that the brightest GFP-expressing stromal cells (commonly referred to as CXCL12-abundant reticular—CAR-cells) are distributed around BM sinusoids [11]. More recently, BM MSC with hematopoietic support activity were also identified by their ability to express Nestin, a type VI intermediate filament protein that is typically found in neuronal progenitors [12,13], while BM MSC with tri-lineage differentiation capacity into osteoblasts, chondrocytes, and adipocytes, were found to express the leptin receptor (LepR) [3]. Leptin is a satiety hormone, primarily secreted from the adipocytes that regulate the appetite, bone mass and insulin secretion from the pancreatic beta cells [14,15]. In the hematopoietic system, hematopoietic stem cells (HSCs) and committed adaptive and innate immune cells, including T-cells, B-cells and monocytes, express leptin receptor (LepR), and respond to circulating leptin [16,17] to induce differentiation and/or maturation [14,16]. LepR is a member of the class 1 cytokine receptor family [18]. Activation of LepR by leptin drives the phosphorylation and activation of the transcription factor Signal Transducer and Activator of Transcription 3 (STAT3), which drives the production of anorexigenic peptides that suppress food intake and increase energy expenditure [19]. LepR expression and activity have been well studied in the hypothalamus region in association with energy homeostasis.

LepR^+^ BM MSCs co-express paired related homeobox-1 (Prx1), PDGFRα, and CD51 but express low Nestin, indicating that they represent a distinct subgroup of MSC [20,21]. LepR^+^ cells are primarily quiescent in nature but start proliferating after irradiation or fracture in the BM. They form all the tri-lineage cells and represent a major fraction of CFU-F. Much like CAR cells, LepR^+^ are predominantly located around BM sinusoids and seem to support the proliferative HSC pool, unlike periarteriolar pericytes expressing the nerve/glial antigen 2 (NG2), which seems to be critical to maintain HSC quiescence [22]. Cxcl12 deletion from arteriolar NG2^+^ cells, but not from sinusoidal LepR^+^ cells, impairs HSC localization in the BM. The deletion of the cell cycle entry activator cytokine SCF in LepR^+^ cells, but not in NG2^+^ cells, leads to a significant depletion of BM HSC [23]. In this review, we are focusing on the unique properties of LepR^+^ MSCs in regulating blood, bone and fat formation in the BM.

## 2. Origin of LepR^+^ Cells in BM

LepR^+^ cells arise in the BM perinatally and over time they are enriched, reaching a frequency of 0.3% of enzymatically dissociated BM cells during young murine adulthood [24,25]. Lineage-tracing experiments indicate that LepR only marks the BM MSC up to 2 months of age in the adult mouse [3], the moment at which LepR^+^ cells start differentiating into the osteoblast, chondrocyte and adipocyte lineages [26]. Differentiating skeletal cells progressively lose LepR expression during the process of differentiation [26]. On post-natal day 6, BM LepR^+^ cells are highly proliferative (around 5.7%) and through aging, their replication rate decreases [27]. Fourteen-day administration of 2-bromodeoxyuridine to mice older than 8 weeks finds that only ~5% of the LepR^+^ stromal cells are proliferative, with only ~0.2% of the LepR^+^ stromal cells in mitosis suggesting that LepR^+^ stromal cells are quiescent in nature [3], with the ability to start proliferating after bone injury or BM myeloablative therapy [3]. 

**Figure 1:**
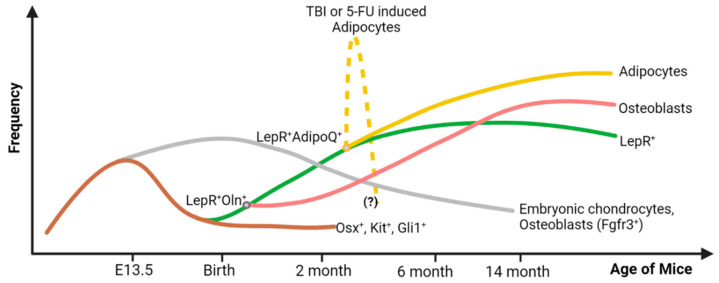
Origin, frequency, and lineage differentiation of the LepR^+^ cell throughout the lifespan of a mouse. During the embryonic stage, the Osx^+^ (and c-Kit^+^Gli1^+^) cells support embryonic bone formation, and their frequency declines rapidly after birth. They are the major source of the embryonic chondrocytes (Fgfr3^+^) that are found in the adult bone as well, but in limited amounts. From these Osx^+^ precursor cells, LepR^+^ cells emerge after birth. They give rise to osteoblasts and adipocytes in adult BM. During hematopoietic crisis due to total body irradiation (TBI) or chemotherapeutic insult (5-FU), a subset of LepR^+^ cells that co-express adiponectin (AdipoQ) give rise to an excessive amount of BM adipocytes. These adipocytes are expected to decrease in number after two or three weeks of 5-FU treatment or TBI, respectively, but this kinetics is not well defined. Figure created with BioRender.com (accessed on 16 May 2024).

Unlike in adult tissues, during embryogenesis, embryonic skeletal stem cells (SSCs) produce embryonic chondrocytes that differentiate into osteogenic and adipogenic LepR^+^ cells post-natally. Currently, we do not know if the emergence of LepR^+^ cells in the BM plays any role in this transition of neonatal hematopoiesis to adult hematopoiesis. Mizoguchi et al. demonstrated that cells expressing Osterix (Osx) are the source of the bone formed during the embryonic stage with a significant decrease in their frequency by 13 weeks of age. Osx^+^ cells also give rise to LepR^+^ cells and Nestin^+^ cells during early post-natal bone development [28] (Figure 1), giving rise to the more mature LepR^+^ cells during the post-natal period [28,29]. Fetal Osx^+^ cells can also express c-kit and BM c-kit^+^ cells, giving rise to at least 20% of adult LepR^+^ MSCs that predominantly form the osteoblasts in the adult mouse [30] (Figure 1). Gli1, another marker for BM MSCs associated with embryonic bone formation, is expressed by the BM MSC of mice up to 1 month of age, when they have hematopoietic supportive activity. After the first month of life, the frequency of BM Gli1^+^ cells declines, giving rise to LepR^+^ cells [31]. Embryonic bone formation primarily happens through Osx^+^ (or Kit^+^ or Gli1^+^) chondrocyte progenitors that later differentiate into osteogenic LepR^+^ cells in the adult stage. Osteogenic LepR^+^ cells play a crucial role in both the maintenance of the bony structure and HSPC trafficking and retention in the BM microenvironment [32], as described later in this review. Together, these data support a paradigm in which different BM MSCs are present in the developing BM and have bone formation and hematopoietic supportive activities at different ontogenic stages.

## 3. LepR^+^ Cells Are Phenotypically and Functionally Heterogeneous

LepR^+^ cells represent the main source of BM osteoblasts and adipocytes in adult BM. With aging, the LepR^+^ keep differentiating to form osteoblasts or adipocytes in the BM. Thus, single-cell transcriptomics always shows a hierarchy of cells that emerge from the LepR^+^ MSCs and then bifurcates into mature adipocytes and osteoblasts. Based on the levels of different adipogenic or osteogenic markers, LepR^+^ cells and their downstream cellular progeny were clustered and further subclustered to define the trajectories of differentiation. Some subsets of LepR^+^ BM MSCs with high Sca-1 expression have higher self-renewal properties [33]. 

LepR^+^ cells have been characterized based on the expression of cytokines and cytokines receptors. High LepR-expressing MSCs that co-express a high amount of SCF reside close to the sinusoids where the long-term HSCs are majorly found [34,35]. These specific high LepR^+^ cells are the ones with higher adipogenic gene expression than others and may differentiate into adipocytes under stress conditions to support newly engrafted HSC/Ps. Green et al. subdivided the BM stromal population expressing PDGFRα and PDGFRβ into four groups as PDGFRα^+^, PDGFRβ^+^, PDGFRα^+^β^+^ and PDGFRα^−^β^−^ cells. These different populations expressed different levels of LepR, with the highest expression of LepR and B-lymphopoiesis-supportive capacity by the PDGFRα^+^β^+^ cell population [36].

Tikhonova et al. performed single-cell (sc) RNA sequencing (RNASeq) analysis to identify different subpopulations of LepR^+^ BM-MSCs in tamoxifen-inducible reporter mice. They identified four different subpopulations of LepR^+^ cells based on the expression of matrix Gla protein (*Mgp*), lipoprotein lipase (*Lpl*), Wnt inhibitory factor 1 (*Wif1*), osteopontin (*Spp1*) and integrin binding sialoprotein (*Ibsp*). These populations were named P1 (*Mgp^High^*), P2 (*Lpl^High^*), P3 (*Wif1^High^*) and P4 (*Spp1^High^Ibsp^High^*). P1 and P2 subsets had higher expression of LepR and adipogenesis-related markers than others. With LepR and ESM1 staining (marker for P1 and P2 populations), they showed that LepR^+^ESM1^+^ cells closely reside within the sinusoids, whereas LepR^+^ESM1^−^ cells reside close to the metaphysis and endosteum capillaries, with predominance near the trabecular region of the bone [37]. P2 and P1 populations expressed higher levels of *SCF*, *CXCL12* and *IL-7*, were enriched in CFU-F, and expressed higher levels of adipocyte lineage genes, compared with P3 and P4 populations [3,38]. Interestingly, they found that after the administration of 5-fluorouracil (5-FU), which eliminates all cycling BM cells in vivo, there is an overall shift towards the adipogenesis-related-marker-expressing cluster with a five-time increase in the size of the P2 population with a concomitant increase in its proliferation. This study also pioneered an adipocytic-primed cluster P5 (*Gas6^High^Hp^High^*) that emerges after 5-FU treatment, which is consistent with the post-myeloablative insult adipocytic expansion in the BM [26,37]. Single-cell RNAseq analysis by Baryawno et al., using a tamoxifen-free approach, which also accounted the bony fragments during the tissue extraction, found that *LepR* expressing MSCs are fundamentally biased towards osteogenic differentiation, and found two clusters with distinct *LepR* expression patterns [38]. The first cluster is formed by LepR^+^ MSCs expressing the highest amount of *LepR* and pre-adipocytic characteristics, the SSC marker Gremlin1 (*Grem1*) [39] and key HSC supporting niche factors (*CXCL12*, *SCF* and *Angiopoietin-1*), but did not express *Nestin* or *Ng2*, while the second cluster is formed by MSC-descendent osteollineage cells (OLCs) that express high levels of *Bglap* (bone gamma-carboxyglutamic acid-containing protein or Osteocalcin) and a moderate expression of *LepR*. Based on the *LepR* expression, *LepR^+^* MSCs were further classified into four subgroups. Out of these, subset 4 of the LepR-MSC group expresses the lowest levels of *LepR* and *SCF* and represents the most differentiated group of MSC with commitment to osteolineage differentiation, forming part of the osteoprogenitor population [38]. Within OLC, they defined the hematopoietic-supporting OLC-1 and the differentiated osteo-chondrocyte lineage OLC-2 populations with potential distinct differentiation origins and with distinct hematopoietic support potential. A major contribution of this report was the description of the subpopulations of LepR^+^ MSC based on the expression of Grem1. Subset 1 of LepR^+^ MSC expresses the highest level of *Grem1* while subset 2 expresses the lowest level. Subset 4 expresses the highest levels of the osteolineage-specific genes Osterix (*Sp7*) and alkaline phosphatase (*Alpl*). All four subgroups of *LepR*^+^ cells have homogenous expression of *Runx2*, another important osteogenic transcription factor [40]. Further analyses of *LepR*^+^ cells during post-natal development described two different groups of *LepR*^+^ MSCs: *LepR^+^ Osteolectin^−^* and (ii) *LepR^+^ Osteolectin*^+^ cells. Of them, *LepR^+^ Osteolectin*^−^ MSCs express the highest level of *SCF* and are enriched in SSC, while *LepR^+^ Osteolectin*^+^ cells express a moderate amount of *SCF* and are osteogenic progenitors [27]. 

Combined, these studies have provided insightful phenotypic and functional information at the single-cell level. Ongoing efforts are focused on the functional characterization of LepR^+^ MSC subpopulations through spatial transcriptomics and other emergent techniques that combine expression, function and microanatomical determinant analyses.

## 4. LepR^+^ Cells Support Hematopoiesis

Hematopoietic stem cells (HSCs), responsible for definitive hematopoiesis, emerge from the hemogenic endothelium of the aorta–gonad–mesonephros region. After that, they move to fetal liver for expansion and at around embryonic day 16.5, they move to the BM, where they get all the spatial microenvironment and niche factors that support the hematopoiesis after birth [1,41]. 

**Figure 2:**
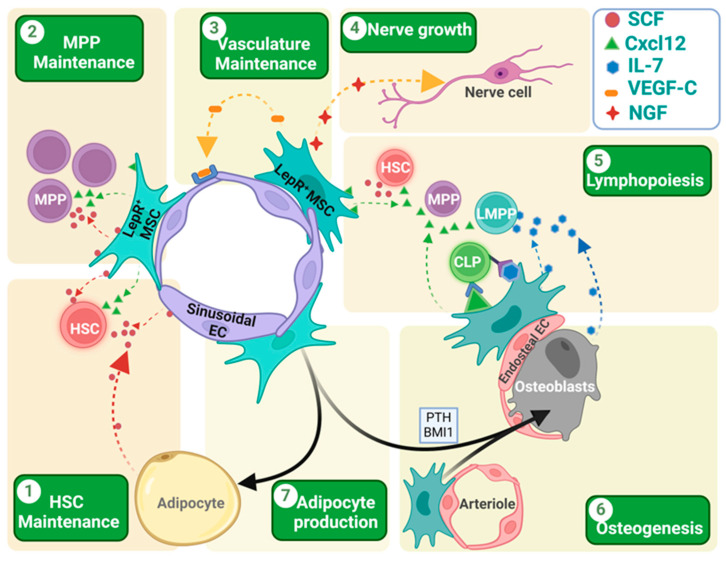
Role of LepR^+^ cells in different aspects of hematopoiesis, osteogenesis and adipogenesis. (1 and 2) LepR^+^ cells express both SCF and Cxcl12, which helps in the HSC/MPP homing, localization, and maintenance. While MPPs are mainly dependent on the SCF produced by LepR^+^ cells, HSCs depend on the SCF produced by both the LepR^+^ cells and sinusoidal endothelial cells. In irradiated mouse BM, newly formed adipocytes become the main source of the SCF, helping in HSC retention and maintenance. (3) LepR^+^ MSCs express VEGF-C that helps in endothelial cell development and vascular microenvironment rebuilding after irradiation-mediated damage in tissue architecture. (4) LepR^+^ MSCs also express the nerve growth factor (NGF) that helps in nerve fiber rejuvenation in BM. (5) LepR^+^ cells play a crucial role in lymphopoiesis. Both LepR^+^ cells and osteoblasts in the endosteal region express IL-7 that helps in lymphocyte differentiation. These IL-7-expressing LepR^+^ cells co-express the membrane-bound Cxcl12 that helps in the close localization of the CLPs for IL-7-mediated induction and differentiation. (6 and 7) LepR^+^ cells are the key source of the osteoblast formation in adult BM. Many factors like the para-thyroid hormone (PTH) or transcriptional factors like BMI1 induce osteoblast formation from the LepR^+^ cells. In the absence of these signals, LepR^+^ cells are destined to become adipocytes. Different arrow colors denote different cell sources. Different chemokines/cytokines are presented with icons depicted in legend inset. Figure created with BioRender.com (accessed on 16 May 2024).

The best-studied mechanism for which LepR^+^ cells support hematopoiesis is their ability to express, present and secrete cytokines and chemokines with potent hematopoietic activity. SCF is one of the important niche factors crucial for the maintenance of HSCs and c-kit^+^ multipotent progenitors that carry out erythropoiesis, myelopoiesis and lymphopoiesis [24,42] (Figure 2). SCF-deficient mice die just after birth due to anemia [20]. Within the BM, LepR^+^ cells, the endothelial cell and adipocytes produce SCF, where LepR^+^ MSCs represent its main source [3,20,27,43]. The LepR^+^ stromal cells are mainly found around the sinusoids and arterioles in mouse BM. It has been noted that peri sinusoidal LepR^+^ cells express higher SCF than periarteriolar cells [3,38], whereas the sinusoidal and arteriolar endothelial cells express a much lower amount of SCF compared to the LepR^+^ cells. Ding et al. demonstrated that SCF from LepR^+^ cells is important in HSC maintenance in the BM. SCF deletion from LepR^+^ cells reduced the BM HSC number, whereas the splenic HSC number was increased, suggesting that, in adult BM, LepR^+^ MSCs are the important source of SCF [20]. Interestingly, c-Kit^+^-restricted multipotent progenitor (MPPs) frequency was also reduced when SCF was deleted from LepR^+^ MSCs, suggesting that MPPs are dependent on the LepR^+^ MSCs [24]. Further, they found that erythroid progenitor differentiation ceased leading to anemia. HSC frequency in the BM was also hampered when SCF was deleted from either the endothelial cell only using Tie2Cre or in double KO mice where SCF was deleted from the endothelial and LepR^+^ MSCs. Interestingly, SCF deletion from endothelial cells did not affect the c-Kit^+^ progenitors, suggesting that SCF from endothelial cells is only used by HSCs, whereas SCF from LepR^+^ cells are shared by HSCs and several downstream MPPs [24]. The importance of the endothelial cells is also suggested by the observation that mouse BM HSCs were reduced in mice with endothelial cell Cxcl12 deficiency [21].

Hematopoietic stress through total body irradiation (TBI) disrupts the BM microenvironment cells that support the hematopoiesis. TBI induces the cell death of larger sets of BM HSC/Ps, LepR^+^ MSCs, and impairs the sinusoidal architecture, reducing the number of sinusoids and VE-cadherin^+^ endothelial cells. Interestingly, unlike sinusoids, TBI seems not to disrupt the arteriolar network significantly [26,44,45,46]. LepR^+^ cells also express the vascular endothelial growth factor (VEGF-C). It acts on the adjacent endothelial cells in a paracrine fashion and helps in their maintenance and growth. After irradiation, LepR^+^ cells increase the expression and secretion of VEGF-C to help endothelial structure repair, and the deletion of VEGF-C from LepR^+^ cells delays the recovery [47] (Figure 2). After myeloablative treatment, there is an expansion of new adipocytes and LepR^+^ cells that express the nerve growth factor (NGF), with positive regulatory activity on nerve fiber growth in the BM (Figure 2). In return, these nerve fibers provide adrenergic neurotransmitters that activate the β2/β3 receptors in the LepR^+^ MSCs and adipocytes to induce the secretion of different hematopoietic niche factors [48].

CXCL12 is a chemokine factor that binds to the CXCR4 receptor and helps in the homing, retention, differentiation and engraftment of different hematopoietic stem and progenitor cells (HSCPs) [49]. Tikhonova et al. showed that LepR^+^ MSC subclusters P1 and P2 have a higher amount of CXCL12 than P3 and P4. However, other reporters showed a comparable level of CXCL12 expression in different subsets at different time points: perinatal day 4, 2 weeks, and 8 weeks. Recently, Schloss et al. showed that the B cell-derived acetylcholine, a parasympathetic neurotransmitter, modulates CXCL12 expression in BM LepR^+^ cells. The inhibition of the acetylcholine signaling increased the inflammatory myelopoiesis [50]. 

The differentiation of lymphoid progenitors depends on both secreted and membrane-bound Cxcl12 and IL-7 (Figure 2). GFP knock-in mice suggest that the IL-7 expressing cells are in the BM parenchyma and endosteal region [51]. Osteoblasts, found mainly in the endosteal region, express moderate levels of IL-7 [52,53], while the LepR^+^ cells there co-express a high level of IL-7 and Cxcl12 [54,55]. The deletion of CXCL12 from the osteoblast using Col2.3-Cre depleted common lymphoid progenitors (CLPs) and lymphoid-primed multipotent progenitors (LMPPs) from the BM, and subsequently reduced the T and B cell in the peripheral blood [21]. Interestingly, there was no change in the HSC frequency or localization. This suggests that a subset of early lymphoid progenitors localizes near the endosteal region. Other mature lymphoid progenitors found close to the perivascular niche depend on the IL-7 from LepR^+^ cells [36,54]. IL-7 deletion from these cells reduced the B cell production [54]. Ebf3^+^/LepR^+^ cells express membrane bound CXCL12. This membrane-bound CXCL12 helps in the retention of the lymphoid progenitors close to the membrane-bound IL-7 and provides positive signaling for B cell production [54,56]. Tissue inhibitors of metalloproteinases (TIMPs) inhibit the activity of matrix metalloproteinase (MMP). The downregulation of TIMPs increases MMP activity, which cleaves the membrane-bound CXCL12 and solubilizes them, and impairs IL-7 signaling and hampers B cell development [57].

Pleiotrophin (PTN) is a neurite outgrowth factor that promotes HSC self-renewal and regeneration in both mice and humans [58]. LepR^+^ MSCs and VE Cadherin^+^ endothelial cells express PTN [59]. The constitutive deletion of PTN from LepR^+^ cells decreased BM HSC content, suggesting the supportive role of LepR^+^ MSC-derived PTN in BM HSC maintenance. In a normal steady-state condition, LepR^+^ MSCs express the highest levels of PTN. However, after total body irradiation, VE Cadherin^+^ ECs increase PTN expression while the PTN expression in LepR^+^ cells is reduced [59]. Another important niche factor secreted by LepR^+^ cells is epidermal growth factor (EGF)-like molecule amphiregulin (AREG). Recently, Wu et al. demonstrated that breast cancer 2 (*BRCA2*) is responsible for DNA damage repair of LepR^+^ cells, and the deletion of this gene increases AREG expression, which in turn induces HSC cycling and exhaustion [60,61]. The LepR^+^ cells also help in platelet homeostasis. It is well known from the work of the Jacobson group that VWF expressing HSCs are platelet/myeloid biased whereas the VWF negative HSCs are lymphoid biased [62,63]. When platelets get activated, they express IL-1, which signals the hematopoietic compartment to replenish platelets. LepR^+^ cells express the IL-1 receptor and further relay the signal to VWF^+^ HSCs to get activated and produce platelets [64]. 

In summary, LepR^+^ cells secrete paracrine factors that regulate every aspect of hematopoiesis, from HSC homing, survival, and regeneration to differentiation, concluding into mature blood cells. They also help in developing the tissue structure where the HSC/Ps reside and regenerate the microenvironment after damage.

## 5. LepR^+^ Cells in Osteogenesis

LepR^+^ MSCs start contributing to adult bone formation after 2 months of age, and by 10 months, they become the main source of osteocytes and bone-lining osteoblasts. Before 2 months of age, only a rare fraction of trabecular osteocytes is formed by LepR^+^ cells, and with age, the trabecular osteocytes are replaced by LepR^+^ cells much faster. By 14 months of age, around 92% of trabecular osteocytes are from LepR^+^ cells, whereas only around 13% of cortical osteocytes are from LepR^+^ cells [3]. Matsushita et al. reported a new SSC with osteoblast–chondrocyte dual identity that helps in bone formation in early life, resides in the endosteal region, and is marked by the expression of fibroblast growth receptor-3 (*Fgfr3*) [65] (Figure 1). Later, LepR^+^ MSCs dominate the osteoblast formation. Shu et al. also described that before adolescence, osteoblasts arise from the chondrocytes, while after adolescence, LepR^+^ cells are the main source of osteoblasts. Periarterial LepR^+^ MSCs are the main source of osteoblast formation and co-express osteolectin (*Oln*). Upon fracture, the LepR^+^Oln^+^ cells get activated and proliferate to produce new osteoblasts. They express a moderate amount of SCF and CXCL12 [27,66]. As stated earlier, the deletion of CXCL12 from either osteoblasts or precursor cells (LepR^+^Oln^+^) did not alter the HSC maintenance or myelopoiesis but reduced the lymphopoiesis [21,66]. This clearly suggests that HSCs and lymphoid progenitors occupy distinct spaces in the BM. The HSCs are suggested to be close to the peri sinusoidal LepR^+^ cells and the lymphoid progenitors are close to the periarteriolar LepR^+^Oln^+^ cells. The work of Baryawno et al. shows that, within the LepR^+^ MSC cluster, the LepR^+^ MSC-4 with lowest expression of *LepR* have the highest *Sp7* expression, denoting an intermediate cell sub-cluster transitioning from LepR^+^ MSCs towards osteoblast differentiation, thus generating the OLC-1 cell cluster. When further sub-clustered, four subtypes of the OLC-1 cell cluster in a continuous lineage were found. Subcluster 1 expresses relatively high levels of *Cxcl12* and *SCF*, along with a moderate level of *LepR*. Meanwhile, subclusters 2–4 lose the *LepR* expression and other niche factor expression, and they start expressing more mature osteolineage markers like *Bglap*, *Spp1* and *CD200* [38]. The authors defined subcluster 4 as a chondrogenic progenitor expressing low levels of the chondrocyte-lineage differentiation transcriptional factor *Sox9* [67] and the chondrocyte secreted extracellular matrix component aggrecan (*Acan*) [68]. However, cartilage formation is mostly embryonic; in adult mice, it is negligible, and there is no significant contribution of LepR^+^ cells in aggrecan^+^ chondrocyte formation in the adult bone in a steady-state condition [3]. Recently, Jeffery et al. found that after bone injury, different populations of SSC form different parts of the bones. In adult BM, a small number of Gli1^+^ cells remain in the periosteal region while the LepR^+^AdipoQ^+^ cells retain in the perisinusoidal space. After a drilling injury, the LepR^+^AdipoQ^+^ cells get activated and form new bones in the trabecular region, whereas bicortical injury activates the Gli1^+^ cells to repair. The Gli1^+^ cells also give rise to the new LepR^+^ stromal cells that express hematopoietic niche factors [69] (Figure 1). Like during post-injury tissue repair, physical loading also helps in bone formation and increases mineralization. Interestingly, physical loading does not induce LepR^+^ cell proliferation or new osteoblast formation, suggesting that LepR^+^ cells are not significantly involved in bone formation after physical loading [25]. Running increases BM LepR^+^ cell frequency, CFU-F generation potential and increased chemokine ligand 2 (CCL2) expression. CCL2 is a major recruiter of LepR^+^ cells towards the bone lining contributing to osteogenesis [70]. 

## 6. LepR^+^ Cell in BM Adipogenesis

For a long time, BM adipocytes have been associated with aging and dysfunctional hematopoiesis. BM adipocytes are different from either white or brown adipocytes in nature [71]. Even different sites of BM contain different types of adipocytes, termed as constitutive and regulatory adipocytes [72,73]. In the unchallenged young mouse, the population of BM adipocytes is low, but with age, their number increases. Interestingly, during adipocyte formation, BM adipocytes lose LepR expression [3]. Recent research suggests their positive impact, at least in stress conditions, when they become the main source of SCF. Perisinusoidal LepR^+^ MSCs are the main source of adipocytes in the BM. As stated earlier, Tikhonova et al. demonstrated that the peri sinusoidal LepR^+^ MSCs express high levels of adipocytic genes and upon chemotherapeutic stress, they increase adipocyte gene expression, proliferate, and give rise to mature adipocytes [37] (Figure 1). These perisinusoidal LepR^+^ MSCs co-express adiponectin (AdipoQ) that represents around 5% of the total LepR^+^ cell. Interestingly, the same LepR^+^AdipoQ^+^ MSCs change their fate towards bone formation after fracture [69]. Zhou et al. demonstrated that these adipocytes are the main source of SCF and help in post-irradiation or post-chemotherapeutic HSC maintenance [26] (Figure 2). In a normal steady-state condition, the adipocytes express SCF; however, their number is very low in young mice (3–5 months old), contributing much less to the BM-SCF level. Under myeloablative stress condition (5-FU chemotherapy or TBI), these LepR^+^AdipoQ^+^ cells transform into adipocytes and increase the BM adipocyte content to support hematopoietic re-establishment (Figure 2). The amount of SCF is higher in the BM microenvironment after chemotherapy or TBI. However, there was no change in the SCF transcript level in the LepR^+^ cells. AdipoQ-specific SCF deletion did not affect the BM SCF level in the steady-state condition, but inhibited the rise in the BM SCF level after TBI, suggesting that the rise in the BM-SCF could be due to the LepR^+^AdipoQ^+^ cells [26]. Interestingly, the adipocytes that appear acutely in response to stress conditions also disappear after the re-establishment of hematopoiesis. Their ultimate fate and mechanism of disappearance remain unknown.

The role of BM adipocytes in hematopoiesis is controversial, probably because of the heterogenic nature of the BM adipocytic populations systemically and regionally within different bone structures. BMAs can create an age-dependent proinflammatory microenvironment and seem to support myeloid-biased blood production. On the other hand, nascent adipocyte populations derived from LepR^+^AdipoQ^+^ MSC differentiation during regeneration after myeloablative therapies seem to be critical for multi-lineage hematopoietic re-establishment. 

## 7. Mechanisms Controlling Fate Decisions of LepR^+^ Cells

LepR^+^ stromal cells sit atop the BM stromal hierarchy and can give rise to either the osteogenic or adipogenic precursor cells. There are many metabolic, hormonal or physiologic factors that regulate the HSC fate decisions. Leptin is mainly expressed by the adipocytes in the body and regulated by the diet condition. It is secreted by adipocytes and is able to regulate the BM MSC differentiation [74]. Different authors reported both the osteogenic and adipogenic role of leptin in BM [75]. Systemic administration of leptin always increases osteogenesis [76]; however, high doses of leptin exert opposite effects. Interestingly, leptin-deficient mice have high adipose tissue and lower bone length, with higher trabecular bone density [77].

To understand how lineage determination happens in LepR^+^ cells, Yue et al. deleted LepR from *Prx1*-expressing cells that include LepR^+^ cells as well as differentiated osteoblasts and chondrocytes. LepR deletion in *Prx1*-expressing cells resulted in mice with normal body mass and normal hematopoiesis, with reduced adipogenesis and increased osteogenesis basally and during repair after injury. LepR signals through Jak2/Stat3 to induce *Cebpa* (CCAAT/enhancer-binding protein alpha) and adipogenesis in the BM [78]. Homeobox (Hox) genes play a critical role in chondrocyte and osteoblastic differentiation; however, different Hox genes are expressed regionally and regulate bone formation in specific bones, defining skeletal diversity. For example, *Hox11* is expressed in the LepR^+^ cells of zeugopod (radius/ulna and tibia/fibula), where it regulates bone formation. *Hox11*-mutated mice exhibit defects in zeugopod formation only, not in the stylopod (humerus and femur) or sternum formation where other *Hox* genes are active [79].

Different metabolic aspects regulate the osteoblast differentiation capacity of LepR^+^ MSCs. For example, it has been reported that in obese people, compared to lean people, (based on body mass index -BMI-), BM-MSCs show increased glycolysis and oxidative phosphorylation with prevalent adipogenic commitment gene expression. They show a more senescence-like phenotype with increased insulin receptors and leptin receptors [80]. Glucocorticoids also have been reported to induce senescence in LepR^+^ SSC, which co express senescence-associated β-galactosidase (SA-βGal^+^) and p16INK4a. The senescence of LepR^+^ cells is a process conducive to osteoporosis. Interestingly, local pulsed electromagnetic fields (PEMFs) [81] and tetramethylpyrazine (TMP) administration [82] can reduce SSC senescence and increase osteogenesis. 

Para thyroid hormone (PTH), an anabolic hormone, is given to patients with osteoporosis [83]. It increases RunX2 expression in LepR^+^ MSCs, which form a multilayered structure near the bone surface. The multilayered cells express Osterix and Type I collagen α, producing mature osteoblasts [84]. Osteolectin is expressed by the LepR^+^ cells, osteoblasts and osteocytes. Upon PTH treatment, LepR^+^ cells increase osteolectin expression, which acts in an autocrine mode of action through the integrin α11 receptor. Osteolectin binding activates the Wnt signaling pathway and helps in osteoblast formation from LepR^+^ cells [85]. PTH also suppresses the expression of *Cebpb*, *Pparg*, and *Zfp467*, a pro-adipogenic zinc finger transcription factor, and increased osteogenic gene expression like *Sp7* and *Col1* in LepR^+^ MSCs [86,87].

Early B cell factor 3 (*EBF3*) is important for maintaining self-renewing state LepR^+^ MSCs. The loss of *EBF3* induces osteogenic differentiation, leading to a reduced LepR^+^ MSC number and HSC niche factors following the occlusion of marrow cavities [88]. Other factors like Erk5 [89] and STAT5a/b [90] are also important for maintaining a normal hematopoietic niche. *Bmi1*, a main component of polycomb repressive complex 1 (PRC1), is critical for repressing different adipogenic genes. When *Bmi1* was deleted from the LepR^+^ cells, osteogenesis was hampered and adipogenesis was promoted. This adipogenic microenvironment failed to maintain HSC/P frequency and induced medullary hematopoiesis [91].

## 8. Conclusions and Future Directions

LepR^+^ cells in the BM are heterogenous and represent a group of cells with pleiotropic effects on the skeletal and hematopoietic activity of bones. Osseous systemic and regional differences in the distribution of LepR^+^ cells and/or their progeny are being evidenced by most recent data. In the adult mouse BM, LepR^+^ cells include mesenchymal stem/progenitor cells and non-stem cells including osteolineage cells, pericytes or endothelial cells, or even, apparently, a fraction of HSCs [17,38]. During repair after myeloablative injury, LepR^+^ cell differentiation is skewed towards adipogenesis through an unclear mechanism that may involve cell-autonomous and non-cell autonomous signaling crosstalk between the surviving BM cells entering division. It is also unclear whether LepR^+^ cells possess similar ability to differentiate into adipocyte formation after myeloablation during the aging process. Thus, further investigations into the origin and regulation of the different types of adipocyte formation in the BM are important. 

## Data Availability

Not applicable.
